# Effect of SiHuangQingXinWan on *Klebsiella pneumoniae*-induced pneumonia: mechanistic insights

**DOI:** 10.3389/fphar.2024.1444439

**Published:** 2024-10-15

**Authors:** Haihui Liu, Xiaoluo Sun, Sian Tao, Shu Liu, Xin Wang, Qiuping Chen, Wenjun Wu, Chongcheng Xi, Baixue Li, Quansheng Feng, Jibin Liu

**Affiliations:** ^1^ School of Basic Medical Sciences, Chengdu University of Traditional Chinese Medicine, Chengdu, China; ^2^ Department of Drug Research and Development, Sichuan Engineering Research Center for Medicinal Animals, Chengdu, China

**Keywords:** SiHuangQingXinWan, *Klebsiella pneumoniae*, pneumonia, omics, anti-inflammatory effect, PI3K/AKT signaling pathway

## Abstract

**Introduction:**

Due to the high mortality rate and increasing severity of antibiotic resistance, there is a growing interest in new treatments for *Klebsiella pneumoni*ae (KP)-induced pneumonia. Research has shown that the single herbs of SiHuangQingXinWan (SHQXW) are effective in treating pneumonia caused by KP. The PI3K/AKT signaling pathway has garnered attention for its potential role in the management of bacterial infections. This study aimed to evaluate the anti-pneumonia effect of SHQXW and to investigate its mechanism of action.

**Materials and Methods:**

The potential plant metabolites and molecular targets of SHQXW in the context of pneumonia were determined through ultra-high performance liquid chromatography-tandem mass-spectrometry (UHPLC-MS/MS) and bioinformatics analysis. The therapeutic effect of SHQXW was evaluated in a KP-induced pneumonia murine model with imipenem/cilastatin as a positive control. Transcriptomics and non-targeted metabolomics were carried out to unveil potential mechanisms and targets for anti-pneumonia effects. Additionally, an in-depth exploration on the PI3K/AKT signaling pathway was conducted in this study.

**Results:**

A total of 24 potential plant metabolites and 285 SHQXW-pneumonia-related targets selected by *Homo sapiens* were identified in this study. The tested doses of SHQXW significantly reduced mortality, improved body weight, decreased the lung index, reduced the bacterial load, and alleviated lung pathological damage in the KP-induced pneumonia murine model (*p* < 0.05). Notably, 1.3 g/kg/day of SHQXW provided the most effective protective outcome. Furthermore, SHQXW demonstrated the ability to suppress the production of inflammatory factors such as IL-1α, IL-1β, IL-3, IL-6, IL-12p70, G-CSF, GM-CSF, MCP-1, KC, and TNF-α. Analysis of transcriptomic and metabolomic data revealed that SHQXW could modulate inflammation-related signaling pathways (TNF, HIF-1, NF-κB, and PI3K/AKT) and metabolites to regulate pulmonary inflammation. Additional experiments using RT-qPCR and western blotting indicated that SHQXW may exert anti-inflammatory effects by activating the PI3K/AKT pathway.

**Discussion:**

The findings indicate that SHQXW effectively reduces inflammation in mice with KP-induced pneumonia by modulating inflammatory signaling pathways and metabolites, rather than by directly inhibiting the growth of KP. This study introduces a novel treatment approach for KP-induced pneumonia and presents a new outlook on drug development.

## 1 Introduction

*Klebsiella pneumoniae* (KP) is a prevalent gram-negative bacteria known for causing hospital-acquired pneumonia (HAP) and ventilator-associated pneumonia (VAP) (Zhang et al., 2014). Additionally, it is commonly pathogen responsible for community-acquired pneumonia (CAP) in Asian countries (Song et al., 2008; Metlay et al., 2019). Pneumonia caused by KP is associated with a significant mortality rate. A study conducted from 2014 to 2016 examined CAP and HAP caused by KP and reported a 28-day mortality rate of 27.9% and 36.9% respectively (Juan et al., 2020). Furthermore, pneumonia-related bacteremia can lead to even higher mortality rates (Forstner et al., 2020). Carbapenems are the cornerstone of therapy for pneumonia caused by KP. These drugs exert their antimicrobial effect by engaging penicillin-binding proteins to impede the assembly of the bacterial cell wall (Cottagnoud, 2002; Wu et al., 2024). However, the genomic plasticity of KP, facilitated by plasmid-mediated transfer and mobile genetic elements, has led to the acquisition of resistance to carbapenems, fostering the proliferation of multidrug-resistant (MDR) and extensively drug-resistant (XDR) phenotypes (Lev et al., 2018). This evolution has positioned KP infections at the forefront of global public health challenges. To counteract these resistance mechanisms, the pharmaceutical industry has advanced novel β-lactam agents, encompassing cefiderocol/sulbactam, ceftazidime/avibactam, meropenem/vaborbactam, and imipenem/relebactam, among others (Doi, 2019). Despite advancements in antibiotic development, the pace lags behind the alarming rate at which resistance is evolving, further exacerbating the challenge of managing infections caused by KP (Wei et al., 2023a).

Faced with the challenges of antibiotic resistance, traditional herbal medicine is gaining prominence for its multifaceted therapeutic profile and immunomodulatory properties. It effectively regulates dysregulated inflammatory responses and immune reactions tied to bacterial infections (Xiang et al., 2024; Zhang et al., 2024a; Zhao et al., 2023). Furthermore, it shows promise in reducing drug resistance, and in certain instances, reversing it, through the inhibition of biofilm development, the suppression of efflux pump gene expression, and the targeting of other drug resistance pathways (Qin et al., 2024; Lin et al., 2024; Meng et al., 2024; Liu et al., 2024a; Wei et al., 2024). As a result, traditional herbal medicine stands out as an innovative therapeutic strategy, warranting further medical research and development. SiHuangQingXinWan (SHQXW), a synergistic herbal blend consisting of *Scutellaria baicalensis*, gypsum, Cortex Phellodendri, *Gardenia jasminoides* Ellis, and rhubarb, has been approved by the China Food and Drug Administration (CFDA) with the National Medicine permission number Z2002014, and by the FDA with an advisory number 2021-0171-A. Given that its single herbs are renowned for their traditional applications in clearing lung heat and detoxification (Luojun et al., 2020), it is hypothesized that this formulation exhibits therapeutic efficacy against pneumonia. SHQXW is based on ancient formulas such as Da Huang Huang Lian Xie Xin Tang and Huang Lian Jie Du Tang. The former is frequently utilized to address infectious diseases like sepsis and pneumonia (Li et al., 2021; Wei et al., 2013). SHQXW has been employed in certain local hospitals in China as a primary or supplementary treatment for infectious diseases such as pneumonia, yielding positive outcomes in ameliorating clinical symptoms. Nonetheless, the antimicrobial properties and underlying mechanisms of SHQXW remain unexplored. The PI3K/AKT signaling pathway, integral to the modulation of inflammatory and immune responses, has garnered attention for its potential role in the management of bacterial infections (Zou et al., 2024; Lovewell et al., 2014; Sun et al., 2013). Traditional herbal medicine is posited to modulate cytokine release and macrophage activity via the PI3K/AKT signaling pathway, thereby potentially contributing to the prevention and management of infectious diseases (Zhang et al., 2024b; Zhang et al., 2022; Hou et al., 2016). The single herbs of SHQXW, such as *S. baicalensis*, have demonstrated the ability to modulate the PI3K/AKT signaling pathway (Cui et al., 2018; Xu et al., 2018). However, the specific influence of SHQXW on this pathway remains an open question that merits further investigation. The exploration of their impact on the PI3K/AKT signaling pathway could unveil novel therapeutic strategies in combating bacterial infections and associated inflammation.

This study employed UHPLC-MS/MS along with bioinformatics analysis to examine the potential plant metabolites and molecular targets of SHQXW. The efficacy of SHQXW against pneumonia was assessed *in vitro* and *in vivo* in KP-infected mice. This study delved into the anti-KP pneumonia mechanism of SHQXW using transcriptomics and metabolomics, and explored the involvement of the PI3K/AKT signaling pathway in the treatment. The results clarify the anti-KP infection mechanism of SHQXW and suggest its promise as a valuable Chinese patent drug for the development of anti-pneumonia medications.

## 2 Material and methods

### 2.1 Reagents and kits

The following reagents were purchased: Tryptone Soya Agar (TSA) (CM0131, Oxoid, United Kingdom), Tryptone Soya Broth (TSB) (CM0129, Oxoid, United Kingdom), imipenem (R843845, seedior, China), imipenem/cilastatin (w039517, Merck Sharp & Dohme Corp, USA), CD86 (GB115630, servicebio, China), CD206 (GB113497, servicebio, China), DAB Kit (G1212-200T, servicebio, China), TRIzol Reagent (15596018, Ambion, United States), the FastPure Cell/Tissue Total RNA Isolation Kit (RC112, Vazyme, China), FAST RT reagent Kit (RR092S, Takara, China), FAST qPCR kit (CN830S, Takara, China), RIPA lysis buffer (BL504A, biosharp, China), PMSF (BL507A, biosharp, China), phosphatase inhibitors (G2007-1ML, Servicebio, China), BCA assay kit (BL521A, biosharp, China), Rabbit antibody anti-PI3K p110a (4249, CST, China), Rabbit anti-AKT antibody (ab179463, Abcam, China), Rabbit antibody anti-phospho-Akt (Ser473) (9271, CST, China), Rabbit anti-β-actin antibody (AF7018, Affnity, China), SHQXW (220401, Shenyang Qing Gong Pharmaceutical Group Co, China). Composition and traditional applications of SHQXW are shown in Table 1.

**TABLE 1:** The composition and traditional applications of SHQXW.

Chinese name	Family	Species	Weight (g)	Part used	Herbal-producing region	Traditional use	References
Rengong Niuhuang	—	Artificial Cow-bezoar	0.015	Synthetic	Hunan	Anti-inflammatory, Antibacterial	Ge et al. (2023), Zang et al. (2011)
Huangbo	Rutaceae	*Phellodendron amurense* Rupr	0.402	Dried bark	Sichuan	Anti-inflammatory, Immunosuppressor	Choi et al. (2014), Mori et al. (1995), Mori et al. (1994)
Huangqin	Lamiaceae	*Scutellaria baicalensis* Georgi	0.402	Dried root	Gansu	Against virus infection, pulmonary infections acute lung injury, Anti-inflammatory Immunological regulatory, Antipyretic, Antibacterial	Wang et al. (2023), Qin et al. (2022), Wei et al. (2023b), Xu et al. (2019) Wang and Li (2023), Zhang et al. (2023), Jiang et al. (2020) Ye et al. (2015), Li et al. (2023) Gu et al. (2023), Zhang et al. (2020)
Dahuang	Polygonaceae	*Rheum officinale* Baill	0.201	Dried root and rhizome	Gansu	Antibacterial, Anti-inflammatory, acute lung injury, Inhibit influenza viruses	Rolta et al. (2020), Babu et al. (2003), Wen et al. (2018), Liu et al. (2022), Lin et al. (2016)
Huashi	Silicate	Talc	0.201	ore	Shandong	Pneumonia	Yang et al. (2021)
Gancao	Fabaceae	*Glycyrrhiza glabra L*	0.268	Dried root and rhizome	Gansu	Anti-pneumonia, Inhibit SARS-CoV-2 infection, Relaxes airway smooth muscle, Immunological regulatory, Antibacterial, acute lung injury, Anti-inflammatory, Antitussive	Jiang et al. (2022), Yi et al. (2022) Liu et al. (2008), Sun et al. (2022) Tanaka et al. (2001) Liu et al. (2020), Chu et al. (2012)
Zhusha	Sulfur compounds	Cinnabar	0.080	ore	Guizhou	Anti-inflammatory	Lin et al. (2006)
Zhizi	Rubiaceae	*Gardenia jasminoides* J.Ellis	0.135	Dried ripe fruit	Jiangxi	Anti-inflammatory Immunomodulatory, Inhibit influenza viruses, bacterial, Pneumonia, Antipyretic	Tian et al. (2022), Shao et al. (2023), Guo et al. (2014) Wu et al. (2021), Zhang et al. (2019)
Shigao	Sulfates	Gypsum	0.268	ore	Shandong	Antipyretic, Antibacterial	Yuan et al. (2002), Behera et al. (2023)
Bingpian	—	Synthetic Borneol	0.028	Processed product	Hunan	Antibacterial, Anti-Inflammatory, Antipyretic	Zhao et al. (2024), Tomaselli et al. (202), Zou et al. (2017)

The plant name has been checked with MPNS (http://mpns.kew.org).

### 2.2 Bacterial strains

KP strain ATCC BAA-1706, procured from the American Type Culture Collection (ATCC), was employed to study bacterial lung infection pathogenesis. It was cryopreserved at −80°C in a solution containing 30% glycerol (vol/vol). For experimental use, the strain was first revived on TSA plates. Then, selected colonies were transferred to TSB liquid medium and incubated for 12 h at 37°C with shaking at 150 rpm. A sub-sample was diluted 1:100 in fresh TSB and incubated for another 10 h at 37°C. The optical density (OD) of the culture at 600 nm was measured, with a value of 0.6 corresponding to a KP concentration of 1.25 × 10^7^ colony-forming units (CFU)/mL.

### 2.3 A preliminary prediction of the potential plant metabolites and their mechanisms of action in SHQXW

The potential plant metabolites in SHQXW and medicated serum were analyzed using ultra-high performance liquid chromatography (UHPLC) coupled with tandem mass spectrometry (MS/MS) on a Q Exactive™ hybrid quadrupole-Orbitrap mass spectrometer interfaced with an Ultimate 3000 RS UPLC system (Thermo Fisher Scientific, Waltham, MA, USA). Potential plant metabolites in SHQXW that had a high mzCloud match (≥ 80) (Liu et al., 2021) were identified and utilized for further analysis. The 2D structure of potential plant metabolites was obtained from the PubChem database (Wang et al., 2017a) and the pharmacokinetic properties of the plant metabolites were evaluated using SwissADME (Daina et al., 2017). The potential targets of the plant metabolites in SHQXW were preliminarily forecasted using the Swiss target prediction database (Daina et al., 2019), the PharmMapper Server database (Wang et al., 2017b) and, the HERB database (Fang et al., 2021). Pneumonia-related gene targets were identified using the GeneCards database (Rappaport et al., 2017), the Comparative Toxicogenomics Database (CTD) (Davis et al., 2021) and the DisGeNET database (Pinero et al., 2020). The common targets between the plant metabolites and pneumonia were determined using Venny 2.1.0. The Gene Ontology (GO) and Kyoto Encyclopedia of Genes and Genomes (KEGG) functional enrichment analyses for all targets was conducted using DAVID database with a screening threshold of *p* < 0.05, count > 3, and false discovery rate (FDR) < 0.05. Subsequently, a drug-disease network diagram was generated using the STRING database and the Cytoscape software. This is a preliminary prediction of the potential plant metabolites and their mechanisms of action in SHQXW. The website of the database is shown in Table 2.

**TABLE 2:** The website of the database used in bioinformatics analysis.

Database	Website
PubChem database	https://pubchem.ncbi.nlm.nih.gov/
SwissADME database	http://www.swissadme.ch
Swiss target prediction database	http://www.swisstargetprediction.ch/
PharmMapper Server database	http://www.lilab-ecust.cn/pharmmapper/
HERB database	(http://herd.ac.cn/
GeneCards database	https://www.genecards.org/
Comparative Toxicogenomics Database	http://ctdbase.org/
DisGeNET database	https://www.disgenet.org/
Venny 2.1.0	https://bioinfogp.cnb.csic.es/tools/venny/
DAVID database	https://david.ncifcrf.gov/
STRING database	https://cn.string-db.org/

### 2.4 KP-induced pneumonia model and therapy

Male BALB/c mice, aged 6–8 weeks, were procured from Beijing HFK Bioscience Co., Ltd. (Beijing, China, approval number SCXK (Jing) 2019-0008). The mice were bred and housed in specific pathogen-free conditions with *ad libitum* access to food and water. The animal procedures in this study adhered to the guidelines for the Use of Laboratory Animals and Institutional Animal Care. Approval for the animal experiments was obtained from the ethics committee of Chengdu University of Traditional Chinese Medicine (authorization number 2022-89). After acclimatization for 1 week, the pneumonia model was established by administering 1.25 × 10^7^ CFU/mL (50 μL per mouse) via tracheal intubation. The blank group received 50 µL of sterile saline through the trachea. A cohort of 10 mice was methodically selected at random from each experimental group for the purpose of conducting survival analysis, while an additional 12 mice per group were designated to investigate molecular changes related to KP-induced pneumonia. There were three treatment groups: the SHQXW-L group was administered 0.65 g SHQXW/kg body weight (half the clinical dose), the SHQXW-M group was administered1.3 g SHQXW/kg body weight (the clinical dose), and the SHQXW-H group was administered 2.6 g SHQXW/kg body weight (twice the clinical dose). Note that the doses of SHQXW-M group are equivalent to the clinical doses for adults, as based on the medication instructions for SHQXW. Imipenem/cilastatin was used as a positive control. The formulation contained an amount of imipenem to cilastatin (1:1, v/v). Following dilution with sterile saline, a dosage of 100 mg/kg body weight/day was administered intraperitoneally. The mice were euthanized via cervical dislocation for further analysis. Survival was assessed using the Kaplan–Meier method. The lung index, calculated as [lung wet weight (g)/body weight (g)] × 100%, was used to assess lung edema. Lung tissue pathology was examined through hematoxylin and eosin (HE) staining. Additionally, lung tissues were frozen in liquid nitrogen and stored at −80°C for further molecular analysis.

### 2.5 Antimicrobial activity evaluation *in vivo* and *in vitro*

Minimal inhibitory concentration (MIC) were carried out by the broth microdilution method in 96-well microtiter plates (Wang et al., 2019) Upon attaining an OD value of 0.6, the bacterial solution was determined to have a concentration of 1.25 × 10^7^ CFU/mL. The solution was diluted to approximately 1.25 × 10^6^ CFU/mL, and 100 μL was mixed with 100 μL of SHQXW or imipenem solution in a 96-well plate, achieving a final bacterial concentration of approximately 6 × 10^5^ CFU/mL. The 96-well tissue culture plates were incubated for 48 h at 37°C. Negative and positive controls were included using TSB and bacterial suspensions without SHQXW or imipenem, respectively. The MIC was determined by measuring the OD at 600 nm after incubation for 48 h. Each MIC assay was repeated at least three times independently, with technical duplicates for each determination. Bronchoalveolar lavage fluid (BALF) samples were collected from the lungs for bacterial counts.

### 2.6 Immunohistochemistry

Formalin-fixed paraffin-embedded lung tissues were cut into 3-μm sections. After antigen retrieval in sodium buffer, the sections were incubated with the appropriate primary antibodies—rabbit anti-CD86 (diluted 1:200) and rabbit anti-CD206 (diluted 1:400), After the primary incubation, the sections were incubated with the appropriate secondary antibody. DAB kit was used to visualize the protein signal. Subsequently, the nuclei were counterstained with Hematoxylin, followed by sealing of the slides and subsequent microscopic examination.

### 2.7 Quantification of cytokines in lung tissue

The multi-plex cytokine analysis on the lung tissuse was analysed by the Luminex 200 system (Luminex Corporation, USA) using cytokine multiplex assays kit (M60009RDP, BioRad, USA). The concentration of each cytokine was normalized per gram of lung homogenate. The following molecules were assessed: interleukins (IL-1α, IL-1β, IL-2, IL-3, IL-4, IL-5, IL-6, IL-9, IL-10, IL-12p40, IL-12p70, IL-13, and IL-17A), colony-stimulating factors (G-CSF and GM-CSF), interferon gamma (IFN-γ), chemokines (MCP-1, eotaxin, MIP-1α, MIP-1β, KC, and RANTES), and tumor necrosis factor alpha (TNF-α).

### 2.8 Metabolomics

Metabolic differences in lung samples from different groups were analyzed by untargeted metabolomics analysis using an ACQUITY UPLC System (Waters, Milford, MA, USA) with Q Exactive (Thermo Fisher Scientific, USA) with ESI ion source (Zelena et al., 2009; Want et al., 2013). Orthogonal projections to latent structures discriminant analysis (OPLS-DA) was conducted for *p*-value, Variable importance projection (VIP). The fold change (FC) was applied to discover the contributable variable for classification. VIP values > 1 and *p* < 0.05 were considered to be statistically significant metabolites. Differential metabolites (DMs) were subjected to pathway analysis by MetaboAnalyst(Pang et al., 2022). The metabolites and corresponding pathways were visualized using the KEGG Mapper tool (Kanehisa et al., 2022).

### 2.9 Transcriptomics and pathway verification

#### 2.9.1 Transcriptomics

Total RNA was extracted from lung tissue using the TRIzol Reagent and quantified with the Agilent 2100 Bioanalyzer system (2100 Bioanalyzer, Agilent, United States). Then, it was used for library construction. Sequencing was performed on a NovaSeq 6000 platform (Illumina) by PANOMIX Biomedical Tech Co., Ltd (Sichuan, China). Differentially expressed genes (DEGs) were identified based on |log_2_FC| > 1 and *p* < 0.05 criteria. Functional enrichment analysis of DEGs included GO and KEGG using eggnog-mapper and the KAAS software (Cantalapiedra et al., 2021; Moriya et al., 2007).

#### 2.9.2 Quantitative real-time PCR

Total RNA was extracted by using FastPure Cell/Tissue Total RNA Isolation Kit. cDNA was synthesized using FAST RT reagent Kit with gDNA Eraser. Quantitative real-time PCR were performed with a real-time PCR system (Bio-Rad). The primers sequences used in this study are listed in Table 3. The relative mRNA expression of PI3K and AKT was calculated by using the 2^−△△CT^ method.

**TABLE 3:** Primer sequences for RT-qPCR.

Gene	Forward primer (5'-3')	Reverse primer (3'-5')
pik3r1	ATT​GAC​AGT​AGG​AGG​AGG​TTG​GAA	TCA​GCC​ACA​TCA​AGT​ATT​GGT​CTC
Akt	CAA​AGG​ATG​AAG​TCG​CCC​ACA	ATC​ACA​AAG​CAT​AGG​CGG​TCA
β-actin	AGA​TTA​CTG​CTC​TGG​CTC​CTA​GC	ACT​CAT​CGT​ACT​CCT​GCT​TGC​T

#### 2.9.3 Western blotting

Murine lung tissue (100 mg) was homogenized in a 2-mL Eppendorf tube with 1 mL of lysis buffer (990 μL RIPA buffer + 10 μL PMSF) and stainless steel balls. After homogenization, the samples were incubated on ice for 30 min and centrifuged at 12,000 rpm for 5 min. The supernatant was collected as the total protein extract. After quantification of protein concentration with a BCA assay kit, 5× loading buffer was added to all samples and heated for 10 min at 100 °C. Protein was separated with SDS-PAGE, and the separated protein was transferred to membranes for western blotting. The primary antibodies were Rabbit antibody anti-PI3K p110a (diluted 1:1000), Rabbit anti-AKT antibody (diluted 1:10000), and Rabbit antibody anti-phospho-Akt (Ser473) (diluted 1:1000), incubated at 4 °C overnight. The secondary antibodies were incubated at room temperature for 2 h.

### 2.10 Statistical analysis

Statistical analysis was conducted using GraphPad Prism 9. It included least squares analysis of variance (ANOVA), a general linear model, and confirmation that the data followed a normal distribution. The data are presented as the mean ± standard deviation (SD). One-way ANOVA and Tukey’s *post hoc* test were employed for comparisons among three or more groups, while Student’s t-test was used for comparisons between two groups. *p* < 0.01 was considered a highly significant difference, and *p* < 0.05 was considered a significant difference.

## 3 Results

### 3.1 Identification of the potential plant metabolites of SHQXW and a preliminary forecast of its mechanism of action

**FIGURE 1:**
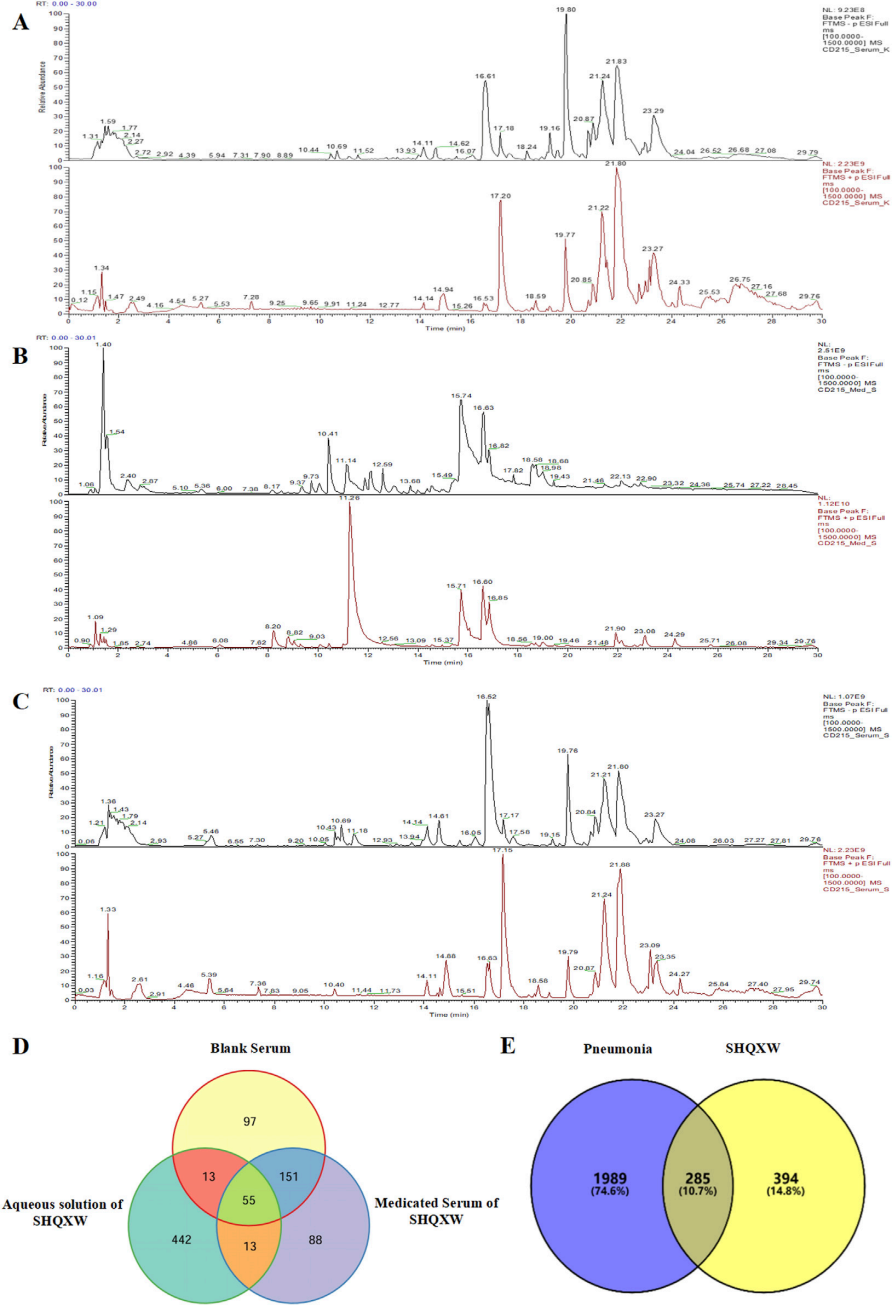
The potential plant metabolites of SHQXW were detected by UHPLC-MS/MS, and their relationship with pneumonia was studied. **(A)** The positive and negative ion mode profiles of normal blank serum establishing a baseline for subsequent comparative analyses. **(B)** The serum positive and negative ion mode profiles after oral administration of SHQXW, revealing the plant metabolites that enter the blood and their ionization characteristics. **(C)** The positive and negative ion mode profiles of the aqueous solution of SHQXW. **(D)** The Venn diagram illustrates the intersection and distinction of potential plant metabolites identified in SHQXW. **(E)** The Venn diagram depicts the relationship between SHQXW and pneumonia, where the purple area represents pneumonia-related entities, the yellow area represents targets of SHQXW, and the intersection indicates the 285 targets (10.7%) common to both, suggesting potential therapeutic targets by which SHQXW could alleviate pneumonia.

The potential plant metabolites in SHQXW were identified through UHPLC-MS/MS (Figures 1A–C). A total of 316 metabolites were identified in blank serum (Serum_K), 307 plant metabolites in medicated serum (Serum_S), and 523 plant metabolites in the aqueous solution of SHQXW (Med_S). Differential analysis of the three datasets using Venny 2.1.0 (Figure 1D) revealed 13 primary plant metabolites and 88 secondary metabolites. These plant metabolites underwent pharmacokinetic and drug similarity evaluation using SwissADME. Among them, 24 plant metabolites that met Lipinski’s rule and any two of the Ghose, Veber, Egan, and Muegge filters, with an mzCloud value ≥ 80, were considered as the potential plant metabolites of SHQXW (Supplementary Table S1).

**FIGURE 2:**
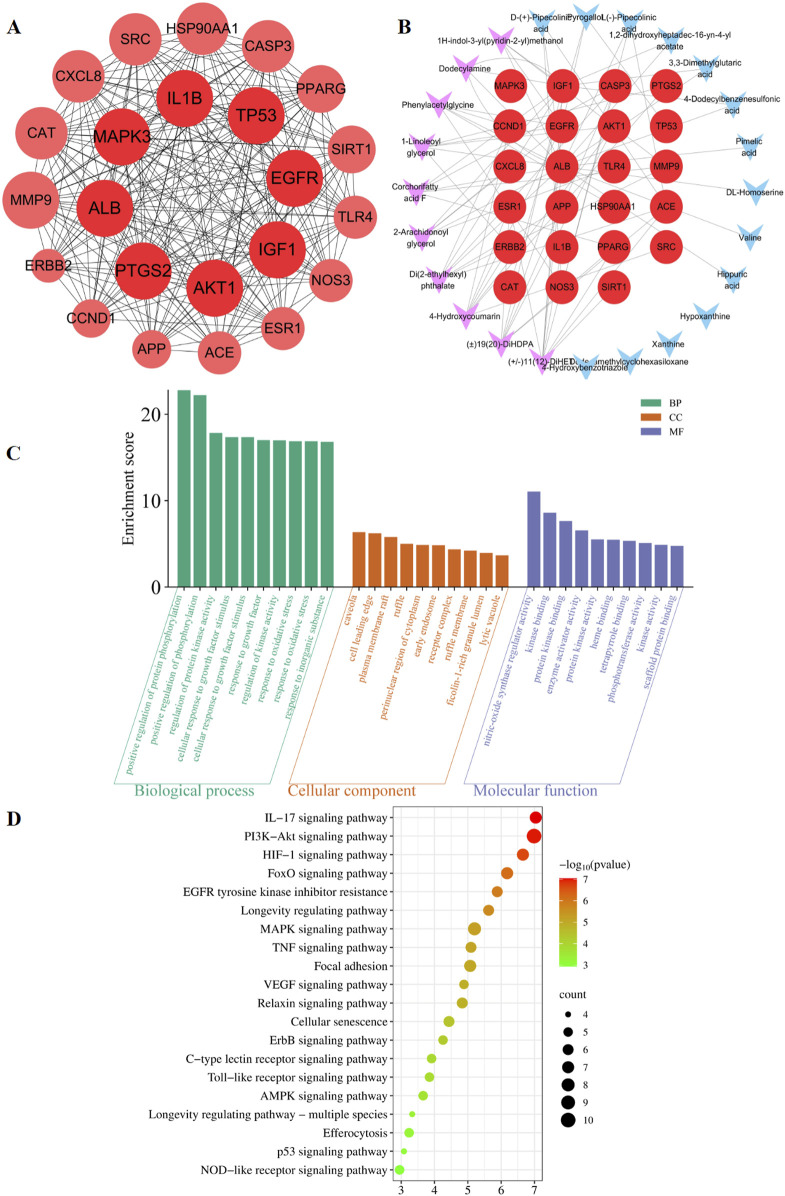
Prediction of the targets and mechanisms of the potential plant metabolites of SHQXW in pneumonia. **(A)** The PPI network shows 23 core targets of SHQXW and pneumonia. **(B)** The plant metabolites-target network diagram illustrates the interplay between the plant metabolites in SHQXW and their corresponding target genes. The core target genes are denoted by red circles, the key plant metabolites from SHQXW are represented by purple V-shaped nodes, and the remaining plant metabolites are indicated by blue V-shaped nodes. **(C)** GO enrichment analysis categorized the biological processes, molecular functions, and cellular components significantly associated with the core targets, providing insights into the physiological roles and pathways influenced by SHQXW. **(D)** KEGG enrichment analysis identified the signaling pathways significantly enriched among the core targets, offering a systematic view of the potential pathways modulated by SHQXW in the context of pneumonia.

The predicted number of target genes for the potential plant metabolites from SwissTargetPrediction, the PharmMapper Server, and HERB was 435, 200, and 134 respectively (Supplementary Table S2–1). A total of 679 gene targets were retained after removing duplicates (Supplementary Table S2–1). The target genes related to pneumonia were collected from GeneCards, CTD, and DisGENET, resulting in 1844, 96, and 963 genes, respectively (Supplementary Table S2–2). After removing duplicates, there were a total of 2274 target genes (Supplementary Table S2–2). Venn diagram revealed 285 common target genes for the potential plant metabolites of SHQXW and pneumonia (Figure 1E). A PPI network was constructed by using STRING and Cytoscape, with average values of degree, betweenness centrality, and closeness centrality of 31.8881, 0.0037, and 0.4806, respectively. The node size was proportional to the degree value. Twenty-three target genes with degree and betweenness centrality values that were twice the average were designated as the core targets (Figure 2A). A plant metabolites-core target network was then constructed, comprising 47 nodes and 77 edges (Figure 2B).

GO and KEGG enrichment analysis was conducted using DAVID (Figure 2C). The GO analysis revealed 388 biological processes, 20 cellular components, and 22 molecular functions (Supplementary Table S3). The KEGG analysis revealed that the core targets are implicated in 115 pathways (*p* < 0.05 and FDR < 0.05) (Supplementary Table S4). Notably, the HIF-1, PI3K-AKT, and IL-17 signaling pathways were among the top 20 pathways potentially linked to pneumonia (Figure 2D). These findings suggest that SHQXW may have a immunoregulatory effect in the context of pneumonia.

### 3.2 SHQXW alleviated mortality and lung injury of mice with KP-induced pneumonia

**FIGURE 3:**
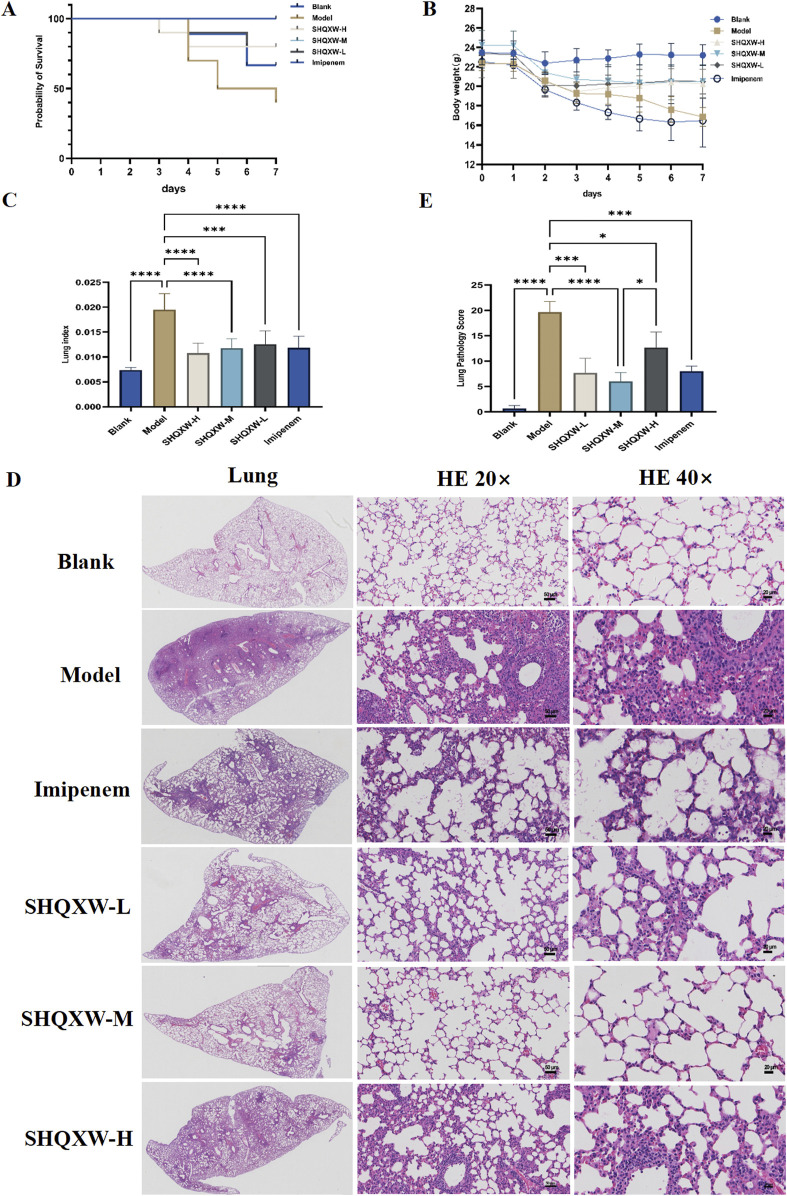
Comprehensive evaluation of the effects of SHQXW on the survival rate, body weight, the lung index, and lung tissue pathology in a KP infection model. **(A)** The Kaplan–Meier survival curves depict the survival rates of mice post-infection with KP and subsequent treatment with SHQXW. **(B)** Analysis of SHQXW’s impact on body weight changes, a common indicator of the health status and the response to infection and treatment. **(C)** The lung index, an indicator of pulmonary edema and inflammation. **(D)** Microscopic examination of HE-stained lung tissue sections, presented at ×20 and ×40 magnification. The eyepiece provides a ×10 magnification. **(E)** Semi-quantitative scoring of lung pathology based on the HE staining results (*p < 0.05, ***p < 0.001, and ****p < 0.0001).

The mice with KP-induced pneumonia presented a 40% survival rate. In contrast, the SHQXW-L, SHQXW-M, and SHQXW-H groups exhibited a survival rate of 80%, 100%, and 80%, respectively, higher than the 70% survival rate for the imipenem/cilastatin group (Figure 3A). Overall, SHQXW improved survival after pneumonia, with the SHQXW-M group exhibiting the most substantial enhancement. Furthermore, the mice with KP-induced pneumonia showed progressive body weight loss (Figure 3B) and a significant increase in the lung index (Figure 3C) compared with the normal mice. SHQXW could reverse the weight loss and decrease the lung index.

The lung injury in the mice with KP-induced pneumonia was evaluated by analyzing HE-stained lung tissue sections that were scored using a standard lung pathology scoring system (Figures 3D, E) (Meng et al., 2022). The mice in the blank group exhibited normal alveolar structure, while those in the model group displayed lung damage such as interstitial thickening, alveolar septal edema, inflammatory cell infiltration, and alveolar wall damage. Treatment with SHQXW effectively reversed the damage caused by KP infection, with significant improvements in the SHQXW-M group. The efficacy of SHQXW to improve lung pathology was comparable to that of imipenem/cilastatin.

### 3.3 The effect of SHQXW on KP *in vivo* and *in vitro*

**FIGURE 4:**
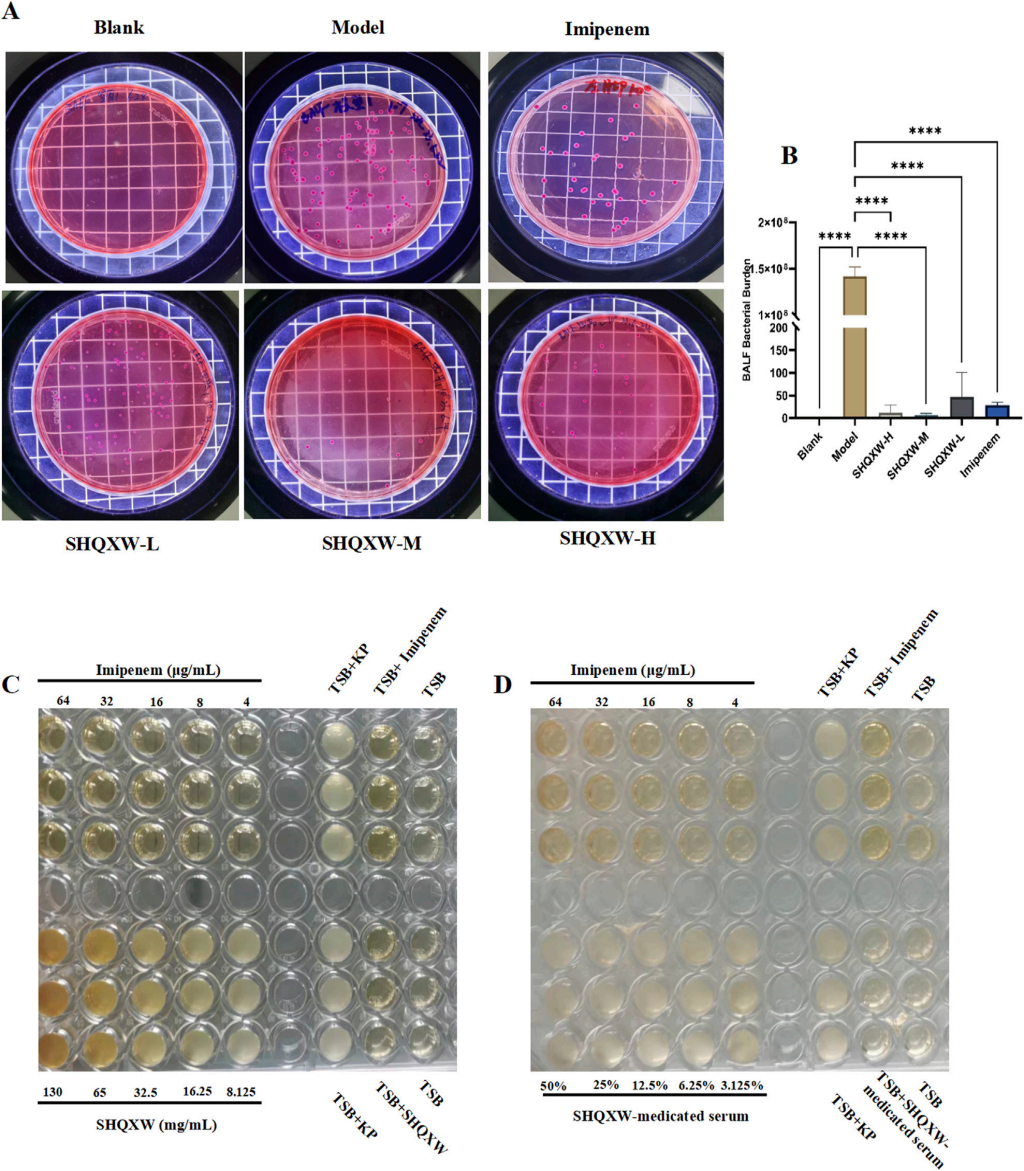
The impact of SHQXW on KP *in vivo* and *in vitro*. **(A)** Quantitative assessment of bacterial load in BALF following treatment, indicating a significant reduction in bacterial counts with SHQXW treatment. **(B)** A comparative histogram of the bacterial load across the study groups. **(C)** The *in vitro* microbroth dilution assay was used to determine the MIC for SHQXW within the range of 8.125–130 mg/mL and for imipenem within the range of 4–64 μg/mL against KP ATCC BAA-1706 in a 96-well plate. **(D)** Evaluation of the antimicrobial activity of SHQXW-medicated serum at a concentration of 3.125%–50% and imipenem at 4–64 μg/mL against KP ATCC BAA-1706 (****p < 0.0001).

To investigate the antibacterial properties of SHQXW and SHQXW-medicated serum against KP, bacterial counts in BALF were evaluated on day 5 post-infection. There was a notable decrease in the counts with SHQXW treatment (Figures 4A, B), and the decrease was more pronounced compared with imipenem/cilastatin. Additionally, the MIC activity was compared with imipenem using the micro-broth dilution method. The MIC assay revealed that both SHQXW (8.125–130 mg/mL) and SHQXW-medicated serum (3.125%–50%) did not demonstrate significant antibacterial activity against KP *in vitro* conditions (Figures 4C, D).

### 3.4 SHQXW inhibited the inflammatory response of mice with KP-induced pneumonia

#### 3.4.1 The effects of SHQXW on macrophages

**FIGURE 5:**
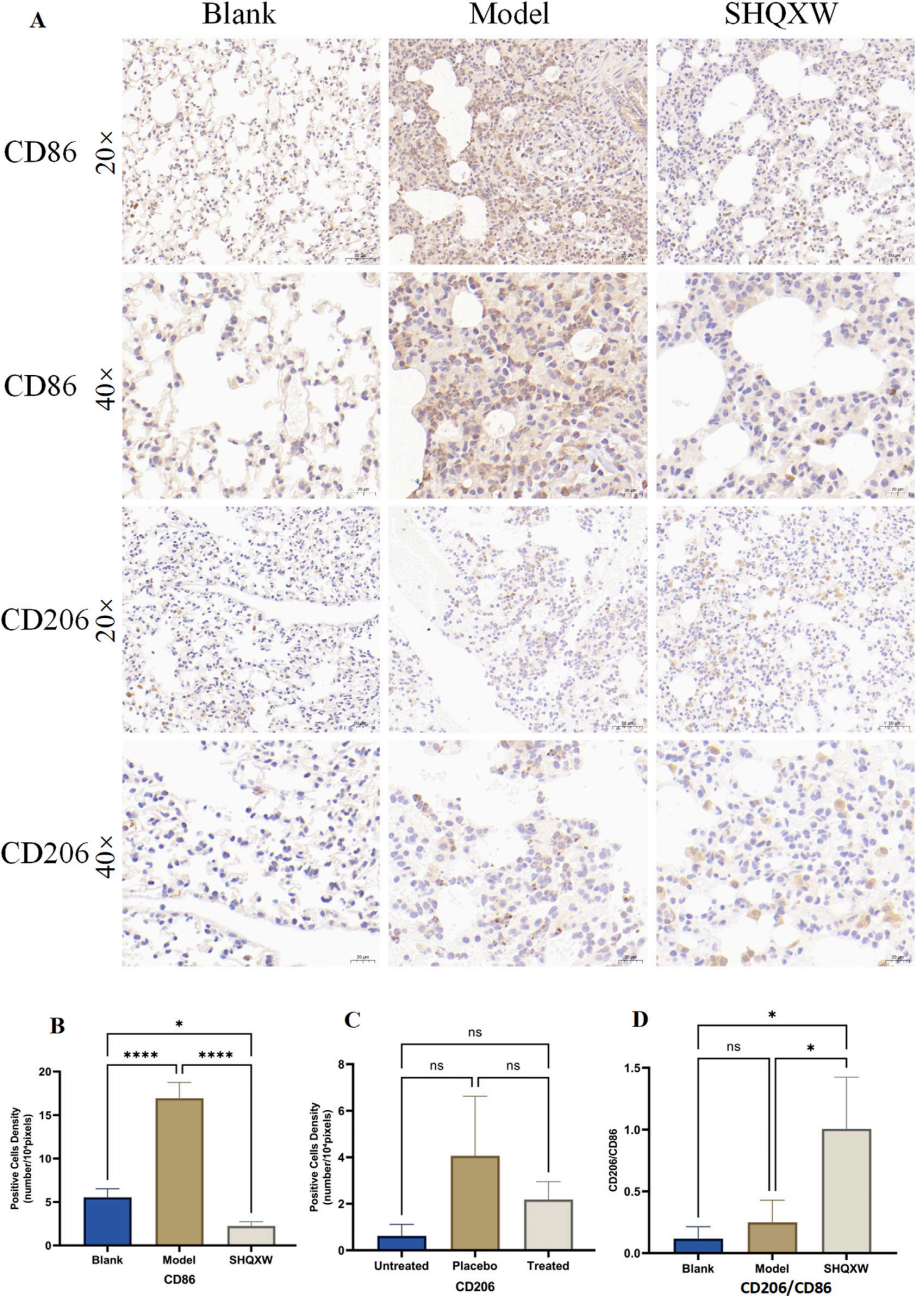
The effects of SHQXW on macrophage changes. **(A)** Immunohistochemical detection of CD86 and CD206 in lung sections, with positive signals indicated by the brown color. The representative images are presented at ×20 and ×40 magnification. The eyepiece provides a ×10 magnification. **(B–D)** Semi-quantitative analysis of immunohistochemical staining for CD86, CD206, and the CD206/CD86 ratio. (*p < 0.05, ****p < 0.0001).

CD86 is commonly used as a marker for M1 macrophages, while CD206 is a marker for M2 macrophages. CD206 and CD86 immunohistochemistry was performed to evaluate the influence of SHQXW on macrophage polarization in lung tissue. Compared with the blank group, CD86 and CD206 expression increased in the model group, but SHQXW treatment reduced these levels. Interestingly, the ratio of M2 to M1 macrophages (CD206^+^/CD86^+^) in the SHQXW group was significantly higher than in the blank and model groups (*p* < 0.05, Figures 5A–D). Hence, SHQXW may play a role in promoting macrophage polarization toward the M2 phenotype.

#### 3.4.2 The effects of SHQXW on inflammatory markers

**FIGURE 6:**
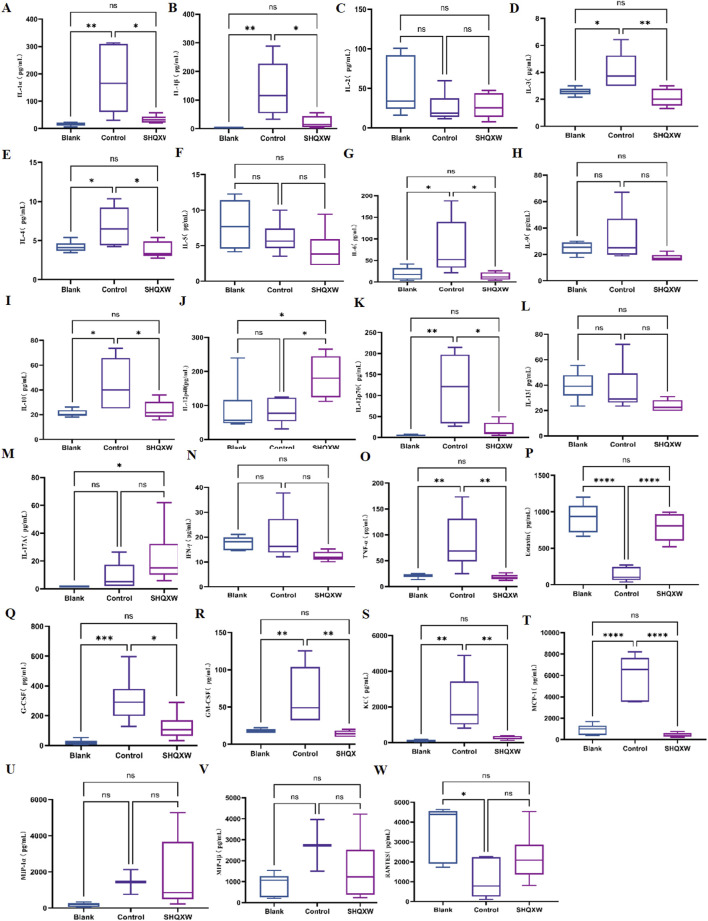
The effects of SHQXW on cytokine expression in the lung tissue. **(A–W)** the concentration of IL-1α, IL-1β, IL-2, IL-3, IL-4, IL-5, IL-6, IL-9, IL-10, IL-12p40, IL-12p70, IL-13, IL-17A, INF-γ, TNF-α, eotaxin, G-CSF, GM-CSF, KC, MCP-1, MIP-1α, MIP-1β, and RANTES. The data were presented as the mean ± SD (n = 6), and were analyzed with one-way ANOVA followed by Tukey’s multiple comparison test (*p < 0.05, **p < 0.01, ***p < 0.001, and ****p < 0.0001).

To assess the inflammatory effects of KP-induced pneumonia, 23 inflammatory markers were analyzed in lung tissue (Figures 6A–W). There was a substantial rise in inflammatory cytokines (IL-1α, IL-1β, IL-3, IL-4, IL-6, IL-10, IL-12p70, G-CSF, GM-CSF, MCP-1, KC, and TNF-α) in the model group compared with the blank group. Conversely, there was a significant decrease in IL-1α, IL-1β, IL-3, IL-4, IL-6, IL-10, IL-12p70, G-CSF, GM-CSF, MCP-1, KC, and TNF-α following SHQXW treatment (*p* < 0.05). The eotaxin and RANTES levels were notably elevated in the SHQXW group compared with the model group (*p* < 0.05). Additionally, IL-12p40 expression was significantly higher in the SHQXW group compared with the blank and model groups (*p* < 0.05). These results suggest that SHQXW treatment can mitigate the systemic inflammatory response in mice with the KP-induced pneumonia.

### 3.5 The effects of SHQXW on the lung metabolome in mice with KP-induced pneumonia

**FIGURE 7:**
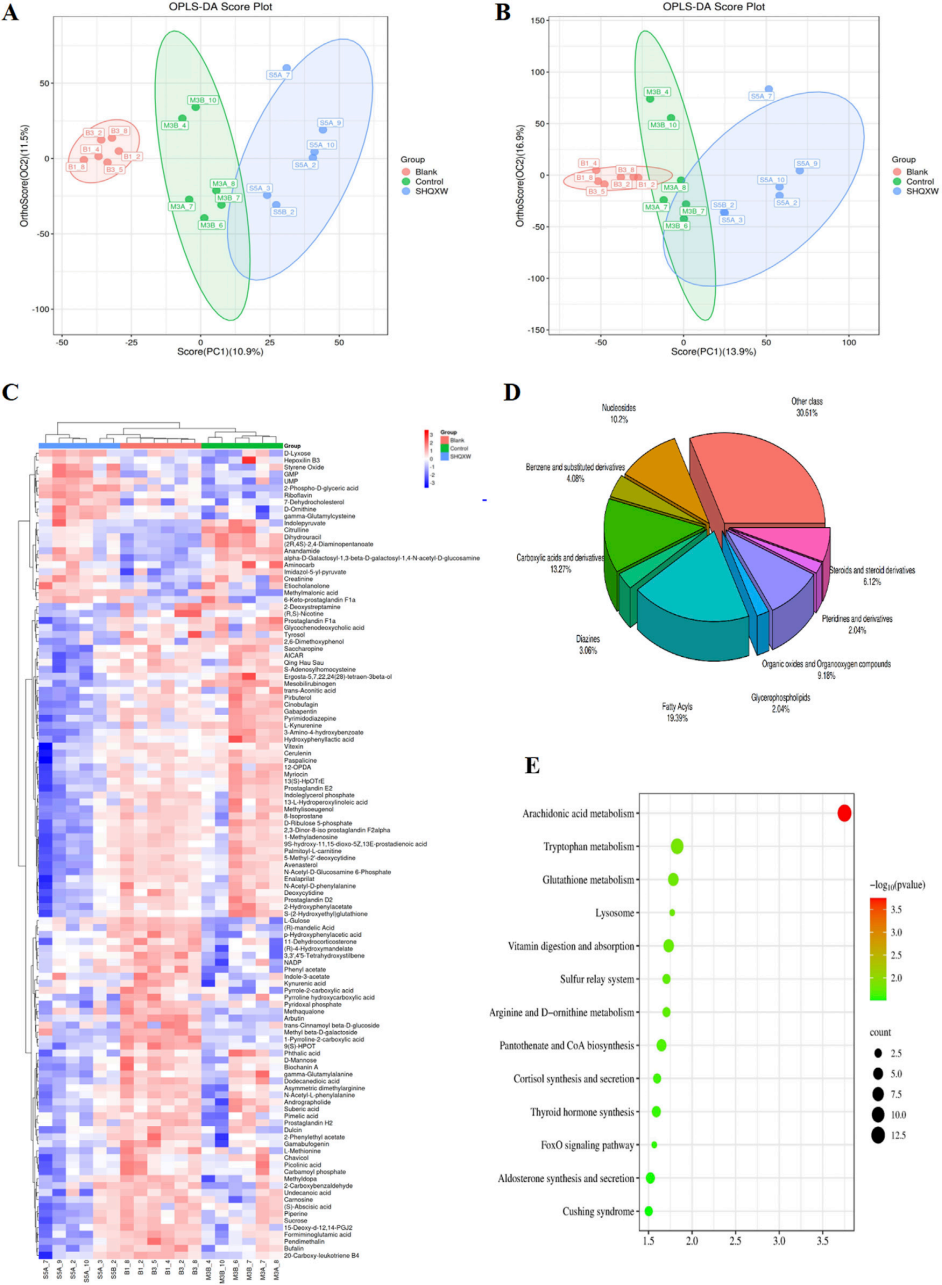
Untargeted metabolomic profiling of Lung tissue derived from the experimental groups (n = 6). **(A, B)**. The OPLS-DA plots, constructed in the positive and negative ionization modes, delineate the metabolite distinctions among the blank, model, and SHQXW groups based on DMs.**(C)** The heatmaps illustrate the metabolite expression patterns and their clustering tendencies across samples from the different groups. **(D)** Classification of DMs restored after SHQXW intervention, indicative of the SHQXW’s restorative effects on the metabolome. **(E)** A map of the metabolic pathways significantly altered following SHQXW treatment, as revealed by a comparative analysis between the model and SHQXW groups.

The impact of SHQXW on the lung metabolome in mice was evaluated with untargeted metabolomics. Quality control samples were grouped together in a central cluster, indicating the stability and reliability of the metabolomics data obtained from LC-MS analysis. OPLS-DA conducted under both positive and negative ion modes revealed distinct differences in the lung metabolic profiles among the three groups (Figures 7A, B). Hierarchical clustering analysis was performed with the relative value of metabolites as the metabolic level, and the results were visualized with heatmaps (Figure 7C). A total of 5,656 primary and secondary metabolites were identified in the lung tissues of all experimental groups (Supplementary Table S5–1). Using a significance threshold of *p* < 0.05 and a VIP score> 1, there were 183 DMs between the blank and model groups, 195 DMs between the model and SHQXW groups, and 159 DMs between the blank and SHQXW groups (Supplementary Table S5–2, 5-3, and 5-4, respectively). Subsequently, a detailed analysis of metabolite changes in lung tissues of KP-infected mice post-treatment was conducted by comparing DMs between the blank and model groups and between the model and SHQXW groups. Metabolites with a log_2_FC ≥ 1 were considered to be upregulated, while those with a log_2_FC ≤ −1 were considered to be downregulated. Compared with the blank group, 122 metabolites, such as 5-keto-D-gluconate, arbutin, leukotriene C4, and uridine, were downregulated (Supplementary Figure S1), whereas 35 metabolites, including dihydrouracil, docosahexaenoic acid, propanoyl phosphate, and sedoheptulose 1,7-bisphosphate, were upregulated in the model group (Supplementary Figure S2). Among the DMs, 98 were restored following SHQXW treatment. These metabolites can be categorized into nucleosides, benzene and substituted derivatives, Carboxylic acids and derivatives, Diazines, Fatty Acyls, Glycerophospholipids, Organic oxides and Organooxygen compounds, Pteridines and derivatives, Steroids and steroid derivatives, as well as other classes (Figure 7D). These data suggest that SHQXW exerts a regulatory effect on these metabolites.

KEGG enrichment analysis of the DMs revealed 13 significantly enriched pathways (*p* < 0.05), namely arachidonic acid metabolism, tryptophan metabolism, glutathione metabolism, lysosome, vitamin digestion and absorption, D-arginine and D-ornithine metabolism, the sulfur relay system, pantothenate and CoA biosynthesis, cortisol synthesis and secretion, thyroid hormone synthesis, the FoxO signaling pathway, aldosterone synthesis and secretion, and cushing syndrome (Figure 7E). Following SHQXW treatment, seven metabolic pathways were differentially regulated, namely arachidonic acid metabolism, vitamin digestion and absorption, glutathione metabolism, sulfur relay system, cortisol synthesis and secretion, the FoxO signaling pathway, and cushing syndrome. SHQXW treatment had the most pronounced effect on arachidonic acid metabolism.

### 3.6 The effects of SHQXW on the lung transcriptome of mice with KP-induced pneumonia

**FIGURE 8:**
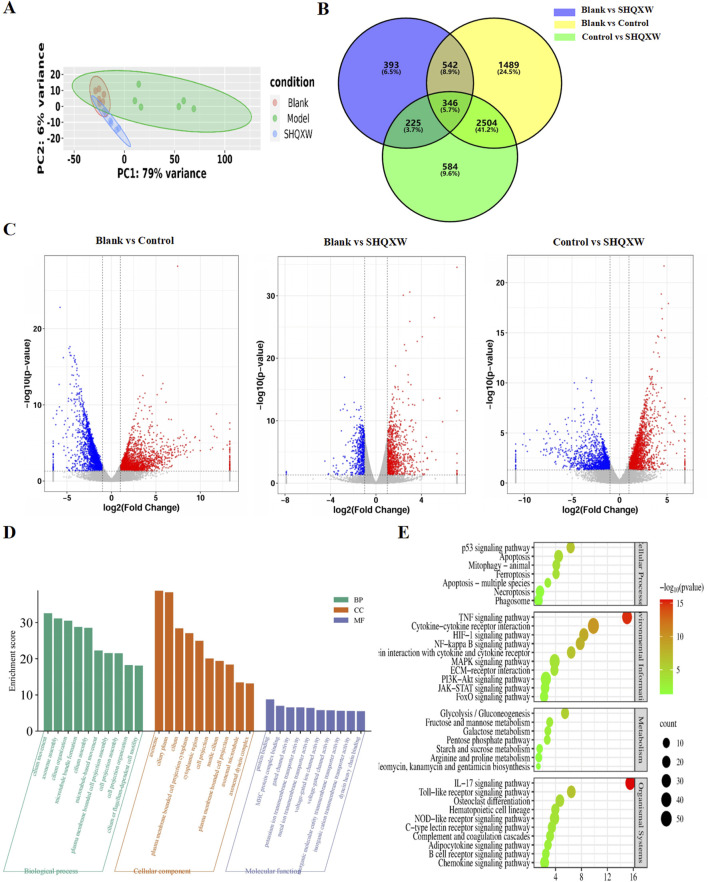
Comprehensive analysis of DEGs in lung tissues from the experimental groups (n = 6). **(A)** The PCA plot illustrates the overall distribution and variance of DEGs across the different groups, providing a snapshot of the gene expression profiles. **(B)** The Venn diagram depicts the unique and intersecting sets of significantly enriched DEGs identified in pairwise group comparisons, highlighting both specific and shared genetic responses. **(C)** The volcano plot shows DEGs where red dots indicate significantly upregulated genes, blue dots signify significantly downregulated genes, and grey dots represent genes with no significant change in expression. **(D)** GO enrichment analysis (for the model vs. SHQXW groups compared with the blank vs. model groups) delineating the functional categories significantly associated with the observed DEGs. **(E)** The enriched pathways in the lung after SHQXW treatment (for the model vs. SHQXW groups compared with the blank vs. model groups). The size of the bubbles corresponds to the number of genes within each pathway; the color intensity of the bubble is inversely related to the *p*-value, indicating the statistical significance of the enrichment.

RNA-sequencing (RNA-Seq) was conducted on lung homogenates to explore the mechanisms by which SHQXW exerts its effects. A total of 21959 genes were identified using next-generation sequencing (NGS) across all samples (Supplementary Table S6–1). DEGs were identified as |log_2_FC| > 1 and *p* < 0.05, and were visualized in a heatmap using hierarchical clustering. The SHQXW group exhibited a similar gene expression patterns as the blank group, but a distinct patterns compared with the model group (Figure 8A). The DEGs in each group are illustrated in a Venn diagram (Figure 8B), and a volcano plot illustrats the overall distribution of DEGs (Figure 8C). Comparison between the blank and model groups revealed 4881 DEGs, of which 2216 were upregulated and 2665 were downregulated (Supplementary Table S6–2, Supplementary Table S6–3). The SHQXW group exhibited 1506 DEGs, including 937 upregulated and 569 downregulated genes (Supplementary Table S6–4, Supplementary Table S6–5). Additionally, there were 3659 DEGs in the SHQXW group compared with the model group, of which 2086 genes were upregulated and 1573 genes were downregulated (Supplementary Table S6–6, Supplementary Table S6–7). Moreover, there were 2850 overlapping DEGs between the model versus SHQXW group and the blank versus model group comparisons, with 1585 DEGs upregulated and 1265 DEGs downregulated Supplementary Figure S3), suggesting a potential crucial role in the alleviation of pneumonia by SHQXW.

The 2850 overlapping DEGs were subjected to GO and KEGG enrichment analysis (Figures 8D, E). GO analysis showed that SHQXW mainly affects cilium organization assembly and movement regarding biological processes and cellular components, and protein binding, gated channel activity, and ion transmembrane transporter activity regarding molecular functions. KEGG analysis revealed that compared with the blank versus model group comparison, SHQXW regulates the TNF, HIF-1, NF-κB, PI3K/AKT, IL-17, and Toll-like receptor signaling pathways; is involved in cellular processes such as apoptosis, mitophagy, ferroptosis, necroptosis; and is involved in metabolic processes including glycolysis/gluconeogenesis, fructose and mannose metabolism, galactose metabolism, the pentose phosphate pathway, starch and sucrose metabolism, arginine and aroline metabolism, and neomycin, kanamycin and gentamicin biosynthesis.

### 3.7 PI3K/AKT pathway involved in the regulation of inflammation by SHQXW

**FIGURE 9:**
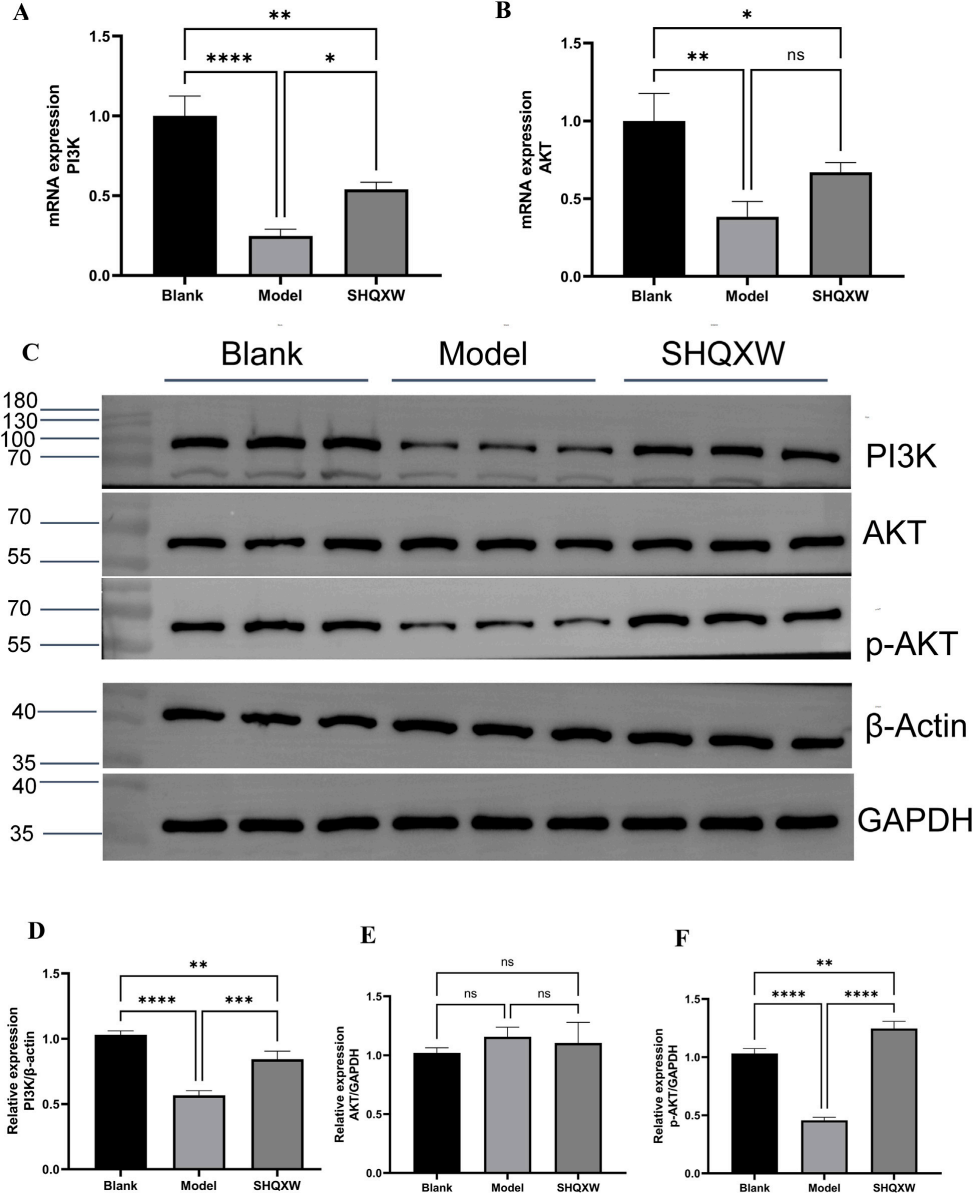
The PI3K/AKT signaling pathway is implicated in the modulatory effects of SHQXW on the inflammatory response. **(A, B)** Quantitative real-time PCR was employed to assess mRNA expression levels of PI3K and AKT across lung tissue samples from all experimental groups. **(C)** Western blotting was conducted to quantify PI3K, AKT, and p-AKT protein expression in lung tissue homogenates from each group. **(D–F)** The histograms present the relative expression of PI3K, AKT, and p-AKT. (**p < 0.01, ***p < 0.001, ****p < 0.0001).

PI3K/AKT is a crucial inflammatory regulatory pathway(Liu et al., 2024b; Jia et al., 2024). To investigate whether SHQXW modulates KP-induced inflammation via the PI3K/AKT pathway, PI3K and AKT gene and protein expression was determined. Western blotting revealed a significant decrease in the PI3K and p-AKT levels in the model group compared with the blank group. Treatment with SHQXW increased the PI3K and p-AKT levels (Figures 9C–F). Moreover, PI3K and AKT gene expression was notably upregulated following SHQXW treatment (Figure 9A, B), suggest that SHQXW may regulate inflammation caused by KP by activating the PI3K/AKT pathway.

## 4 Discussion

In recent decades, KP, especially MDR strains, has become a major cause of infections acquired in hospitals and communities throughout the world. The need for new therapeutic approaches to combat this issue is evident. Immunotherapy has shown potential as an effective strategy against these superbugs. In addition, passive immunization against KP has been extensively studied as an immunotherapy method. (Robert et al., 1982; Yoshida et al., 1981; Takahashi et al., 1984). Traditional herbal medicine has been proposed as an alternative treatment for KP infections, as it not only directly inhibits bacteria but also regulates the immune response in the host. SHQXW is a prescription medicine that has been approved by the China Food and Drug Administration (CFDA) with the National Medicine approval number Z2002014, and it has also been approved by the FDA with an advisory number 2021-0171-A. The adult dosage of SHQXW as stipulated in the drug instructions is 10 g per day. Consequently, the dosage for adult use is converted to a dosage for mice based on body surface area (BSA) normalization method according to the instructions for SHQXW(Reagan-Shaw et al., 2008). Given the higher metabolic rate of rodents, the study established three dosage gradients (SHQXW-L: 0.65 g SHQXW/kg body weight (half the clinical dose), SHQXW-M: 1.3 g SHQXW/kg body weight (the clinical dose), SHQXW-H: 2.6 g SHQXW/kg body weight (twice the clinical dose)) on the basis of the calculated dosage to more precisely explore the optimal dosage. In the present study, *in vivo and in vitro* assays were conducted to assess the antibacterial effects of SHQXW. Interestingly, SHQXW did not show a bacteriostatic effect against KP -induced pneumonia in either setting, but it did improve the survival rate, reduce the lung bacterial load, and promote recovery of various health indicators. Particularly, the effects in the SHQXW-M group were the most pronounced, aligning with the adult dosage specified in the instructions. This suggests that SHQXW may achieve therapeutic effects through pathways other than direct bacterial inhibition. Further research has revealed that SHQXW also suppresses the expression of the pro-inflammatory cytokines IL-1α, IL-1β, IL-3, IL-6, TNFα, G-CSF, GM-CSF, and MCP-1, which are closely associated with macrophages. Macrophages release IL-1, IL-6, TNFα, MCP-1, G-CSF, and GM-CSF in response to microbial infections. Additionally, IL-3 and GM-CSF can enhance cytokine production by macrophages, while MCP-1 aids in the recruitment of circulating monocytes to tissues. These cytokines play a role in modulating various macrophage functions and cell surface marker expression (Cavaillon, 1994). Macrophages are pivotal in the inflammation and tissue damage induced by gram-negative bacteria such as KP (Cheng et al., 2021). They can differentiate into the M1 or M2 phenotype (Hoh et al., 2019). When infected by external pathogens like KP, macrophages tend to polarize toward the M1 phenotype and release pro-inflammatory cytokines such as TNF-α, IL-1β, IL-6, and IL-12 (Hirani et al., 2022; Guimaraes et al., 2022). However, excessive M1 polarization and activation of inflammation can result in tissue damage (Chen et al., 2021). On the other hand, shifting macrophages toward the M2 phenotype can help reduce lung inflammation and support tissue repair (Ivashkiv, 2013; Aggarwal et al., 2014; Murray et al., 2014). Nevertheless, excessive M2 polarization may lead to the survival of bacteria within macrophages, thereby evading immune clearance (Tian et al., 2023). Therefore, a moderate level of M2 macrophages is conducive to the recovery from inflammation and resistance to bacteria (Chen et al., 2022). In the present study, immunohistochemistry revealed that compared with the blank group, the model group had an increase in CD86 expression, indicating that KP can promote M1 polarization. The model group also showed a significant increase in CD206 expression, indicating that KP can also promote M2 polarization. This finding is consistent with some literature reports: KP can induce excessive activation of M2 macrophages, which may lead to immune evasion (Benoit et al., 2008). Macrophage polarization is a highly dynamic and complex process, where the M1 and M2 phenotypes represent the simplified extremes of a dynamic, continuous spectrum of macrophage changes. Macrophages can shift their phenotypes between these two extremes in response to the ever-changing microenvironmental signals, allowing them to adjust their functional programs according to the varying demands of the tissue repair process (Yan et al., 2024; Anders et al., 2019; Hourani et al., 2023; Ordaz-Arias et al., 2022). The dynamic interaction between M1 and M2 macrophages is crucial for coordinating effective immune responses, balancing inflammation and tissue repair, and maintaining homeostasis within the body. An imbalance in this equilibrium can lead to the development and progression of various pathological conditions, including chronic inflammatory diseases, autoimmune diseases, and impaired wound healing (Kang et al., 2022; Kuo et al., 2021; Feng et al., 2021). Therefore, variation in the M1/M2 ratio, rather than the levels of M1 or M2 macrophages, has more practical significance (Zhang et al., 2024; Huang et al., 2024). In this study, SHQXW reduced both markers after treatment, indicating that it can inhibit excessive M1 and M2 polarization. However, the CD206/CD86 ratio increased significantly with SHQXW, so that treatment seems to be more inclined to promote moderate M2 polarization, which is beneficial for the recovery from inflammation. Hence, SHQXW may have therapeutic effects by balancing anti- and pro-inflammatory factors and by modulating macrophage polarization.

Due to the multi-component, multi-pathway, and multi-target characteristics of traditional herbal medicine, a comprehensive multi-omics analysis was conducted to investigate the mechanism(s) of action of SHQXW. Transcriptomics identified the TNF, PI3K/AKT, HIF-1, and NF-κB signaling pathways as the primary targets of SHQXW in treating KP-induced pneumonia. These results are consistent with those obtained from the combination of UHPLC-MS/MS and bioinformatics analysis. The PI3K/AKT signaling pathway plays a significant role in the regulation of inflammatory responses, although the results have been equivocal. Some studies suggest that the PI3K pathway aids in the activation of NF-κB, thereby promoting inflammation, while others indicate that PI3K may suppress inflammatory responses (Hazeki et al., 2007; Fukao and Koyasu, 2003). The outcome of PI3K activation downstream of immune receptors depends on the type of activated cell. Specifically, PI3K activation significantly inhibits inflammatory responses in monocytes, macrophages, and dendritic cells, but enhances the immune responses of mast cells and possibly other granulocytes (Troutman et al., 2012). Furthermore, PI3K activation is indispensable for promoting immune responses through its ability to promote the survival and proliferation of B and T lymphocytes (Troutman et al., 2012). In the present study, after SHQXW treatment, PI3K and p-AKT expression increased. In conjunction with the changes observed in macrophages in this study, it is hypothesized that macrophages are the primary target cells of this pathway. KP may influence macrophage polarization (Zhao et al., 2021; Yan et al., 2023) by inhibiting the PI3K/AKT pathway (Kamaladevi and Balamurugan, 2017), leading to inflammation. SHQXW may be a crucial factor in the treatment of KP infection through its regulation of the PI3K/AKT signaling pathway. Additionally, HIF1 plays a role in macrophage plasticity (Takeda et al., 2010) and is implicated in the defense against KP by modulating cytokines like TNF (Otto et al., 2021; Holden et al., 2016). Metabolomics employs high-throughput techniques to examine the levels of endogenous metabolites in different mice groups (Clish, 2015). This study revealed that SHQXW has the ability to modulate endogenous metabolic changes in mice with KP-induced pneumonia, particularly affecting the arachidonic acid metabolism pathway. This pathway is closely associated with lung inflammation (Alba-Loureiro et al., 2004; Das, 2021; Ruan et al., 2022), and can influence M1/M2 polarization (Zhou et al., 2023) as well as the phagocytic and bactericidal activity of lung macrophages against KP (Bailie et al., 1996). As a result, it is hypothesized that SHQXW ameliorates KP lung infection by suppressing inflammation and modulating the body’s immune response.

This study provides a preliminary exploration of the regulatory mechanisms of SHQXW in treating pneumonia, utilizing a combination of UHPLC-MS/MS, bioinformatics, transcriptomics, and metabolomics. While this study has enhanced the understanding of the therapeutic effects and mechanisms of action of SHQXW, there are areas that require further investigation. Moving forward, breakthroughs and innovations in research approaches, technical methods, and theoretical modeling are essential to fully uncover the precise therapeutic mechanisms by which SHQXW protects against KP infection.

## 5 Conclusion

This study illustrated that SHQXW could effectively alleviate KP-induced pneumonia in mice by reducing inflammation. In the three dosing groups of SHQXW, the SHQXW-M group demonstrated the optimal therapeutic effect, which aligns with the adult dosing recommendations in the drug instructions of SHQXW. The mechanism involves modulation of inflammatory signaling pathways and metabolites, rather than direct inhibition of KP growth. The integration of bioinformatics, transcriptomics, and metabolomics has provided a comprehensive understanding of the potential therapeutic mechanisms by which SHQXW can alleviate pneumonia.

## Data Availability

The original sequencing data for all transcriptomics samples have been deposited into the NCBI Sequence Read Archive (SRA) public database, with the accession number PRJNA1168868. The remaining datasets presented in this study can be found in online repositories. The names of the repository/repositories and accession number(s) can be found in the article/Supplementary Material.

## Abbreviations

KP, *Klebsiella pneumoniae*; SHQXW, SiHuangQingXinWan; HAP, hospital-acquired pneumonia; VAP, ventilator-associated pneumonia; CAP, community-acquired pneumonia; MDR, multi-drug resistant; UHPLC-MS/MS, ultra-high performance liquid chromatography-tandem mass-spectrometry; TSA, Tryptone Soya Agar; TSB, Tryptone Soya Broth; BP, biological processes; CC, cellular components; MF, molecular functions; MIC, minimum inhibitory concentration; NGS, next-generation sequencing; DEGs, differentially expressed genes; OPLS-DA, Orthogonal projections to latent structures discriminant analysis; HE, hematoxylin-eosin; BALF, bronchoalveolar lavage fluid; CFU, colony forming units; GO, Gene Ontology; KEGG, Kyoto Encyclopedia of Genes and Genomes; VIP, Variable importance projection; FC, fold change; DMs, Differential metabolites; SD, standard deviation; ANOVA, analysis of variance.
