# Supplementary material for: Effect of SiHuangQingXinWan on Klebsiella pneumoniae-induced pneumonia: mechanistic insights
Source: Front Pharmacol. 2024 Oct 15;15:1444439. doi: 10.3389/fphar.2024.1444439 (PMC11519414; doi:10.3389/fphar.2024.1444439)
Supplement: Supplementary file 6 [file DataSheet2.docx]

Object IDs and corresponding URLs:

RUN:36904487: https://www.ncbi.nlm.nih.gov/sra/RUN:36904487

RUN:36904486: https://www.ncbi.nlm.nih.gov/sra/RUN:36904486

RUN:36904485: https://www.ncbi.nlm.nih.gov/sra/RUN:36904485

RUN:36904484: https://www.ncbi.nlm.nih.gov/sra/RUN:36904484

RUN:36904483: https://www.ncbi.nlm.nih.gov/sra/RUN:36904483

RUN:36904482: https://www.ncbi.nlm.nih.gov/sra/RUN:36904482

RUN:36904481: https://www.ncbi.nlm.nih.gov/sra/RUN:36904481

RUN:36904480: https://www.ncbi.nlm.nih.gov/sra/RUN:36904480

RUN:36904479: https://www.ncbi.nlm.nih.gov/sra/RUN:36904479

RUN:36904478: https://www.ncbi.nlm.nih.gov/sra/RUN:36904478

RUN:36904477: https://www.ncbi.nlm.nih.gov/sra/RUN:36904477

RUN:36904476: https://www.ncbi.nlm.nih.gov/sra/RUN:36904476

RUN:36904475: https://www.ncbi.nlm.nih.gov/sra/RUN:36904475

RUN:36904474: https://www.ncbi.nlm.nih.gov/sra/RUN:36904474

RUN:36904473: https://www.ncbi.nlm.nih.gov/sra/RUN:36904473

RUN:36904472: https://www.ncbi.nlm.nih.gov/sra/RUN:36904472

RUN:36904471: https://www.ncbi.nlm.nih.gov/sra/RUN:36904471

RUN:36904470: https://www.ncbi.nlm.nih.gov/sra/RUN:36904470
