# Supplementary material for: Effect of SiHuangQingXinWan on Klebsiella pneumoniae-induced pneumonia: mechanistic insights
Source: Front Pharmacol. 2024 Oct 15;15:1444439. doi: 10.3389/fphar.2024.1444439 (PMC11519414; doi:10.3389/fphar.2024.1444439)
Supplement: Supplementary file 8 [file DataSheet1.docx]

**Supplementary Figures**

**
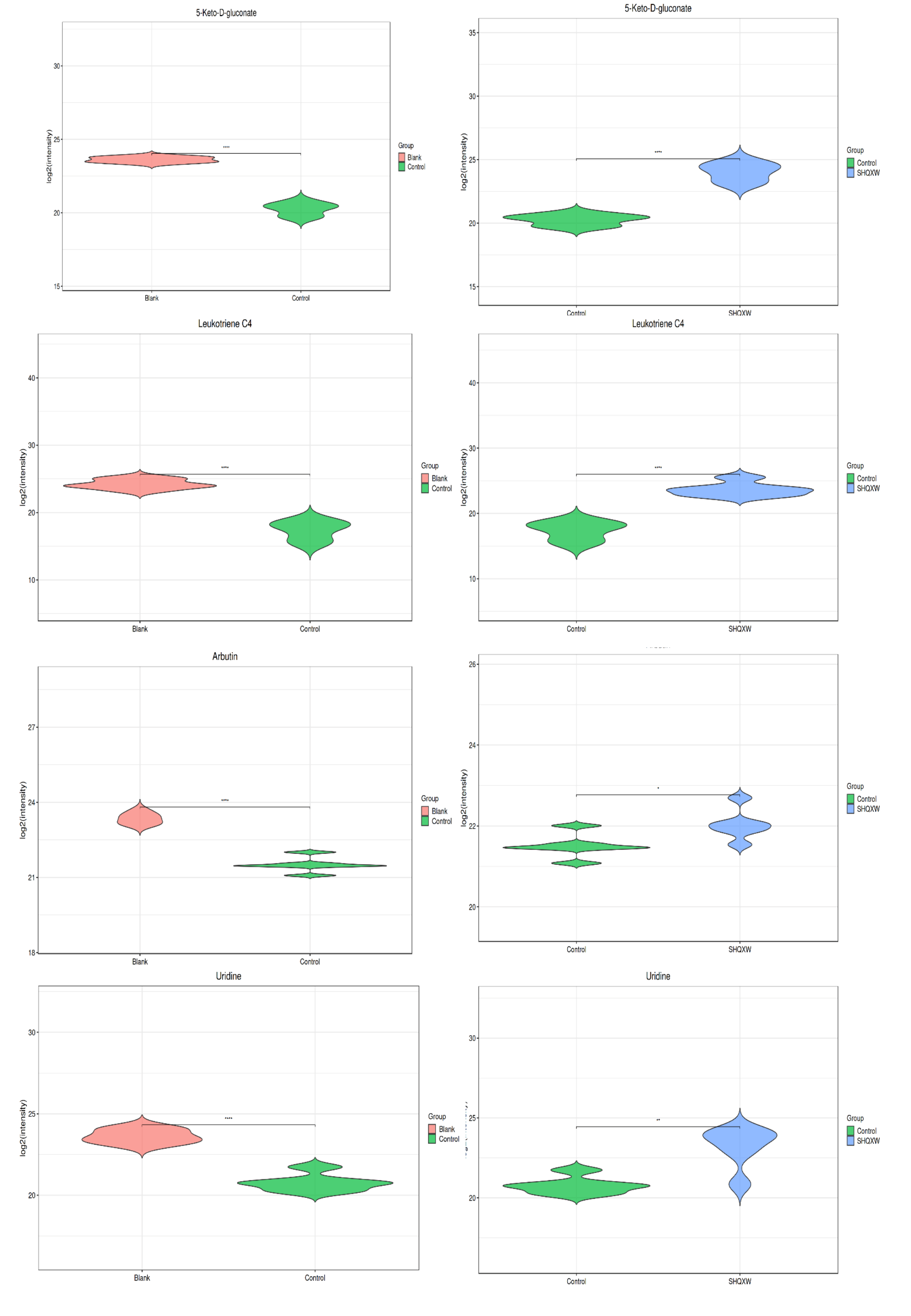
**

**Fig. S1** Representative metabolite changes between the blank group and the model group, as well as between the model group and SHQXW. The blank group was represented by pink, the model group by green, and the SHQXW group by blue. Metabolites were down-regulated in the model group and up-regulated after SHQXW intervention.

**
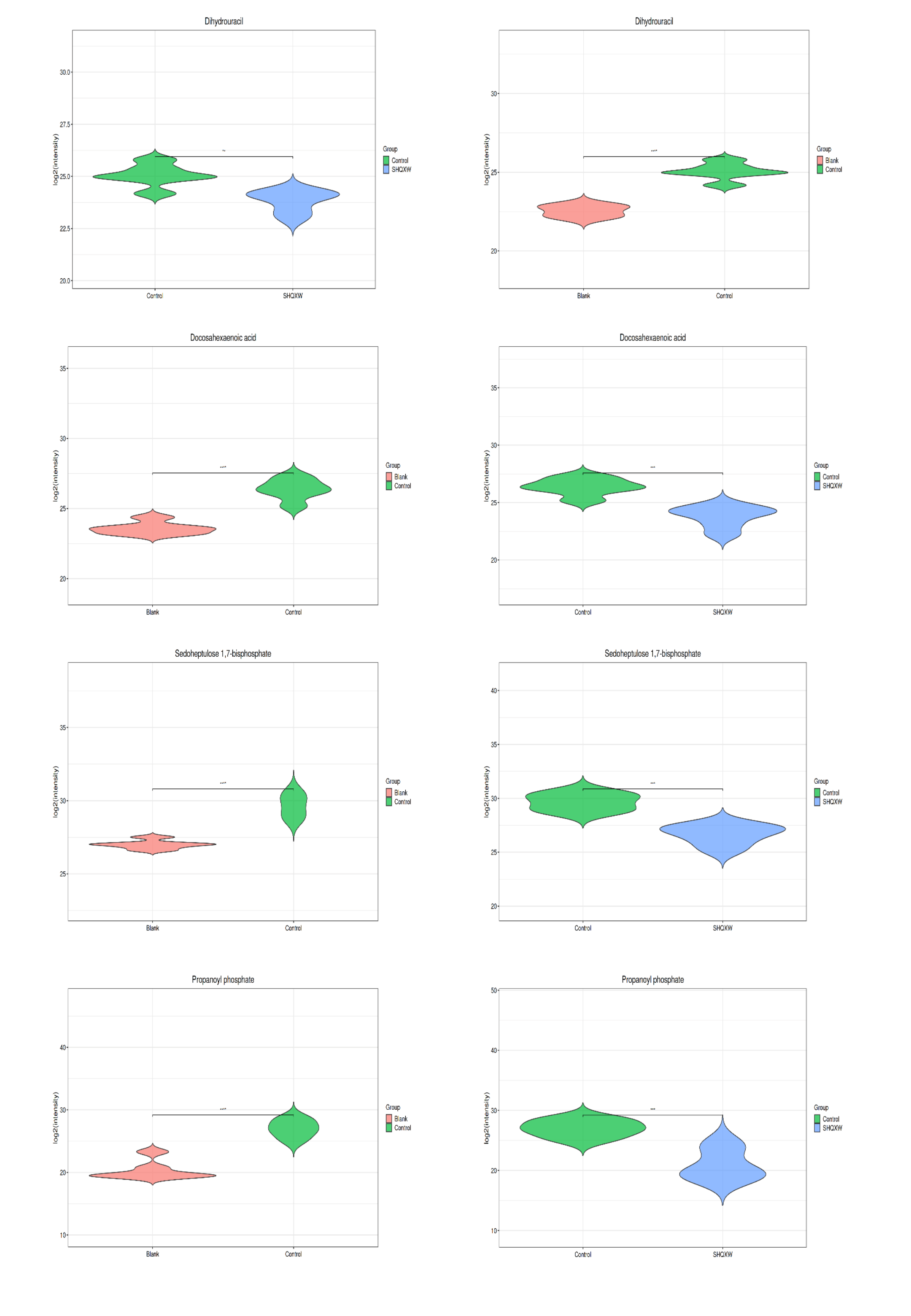
Fig. S2** Representative metabolite changes between the blank group and the model group, as well as between the model group and SHQXW. The blank group was represented by pink, the model group by green, and the SHQXW group by blue. Metabolites were up-regulated in the model group and down-regulated after SHQXW intervention.

**
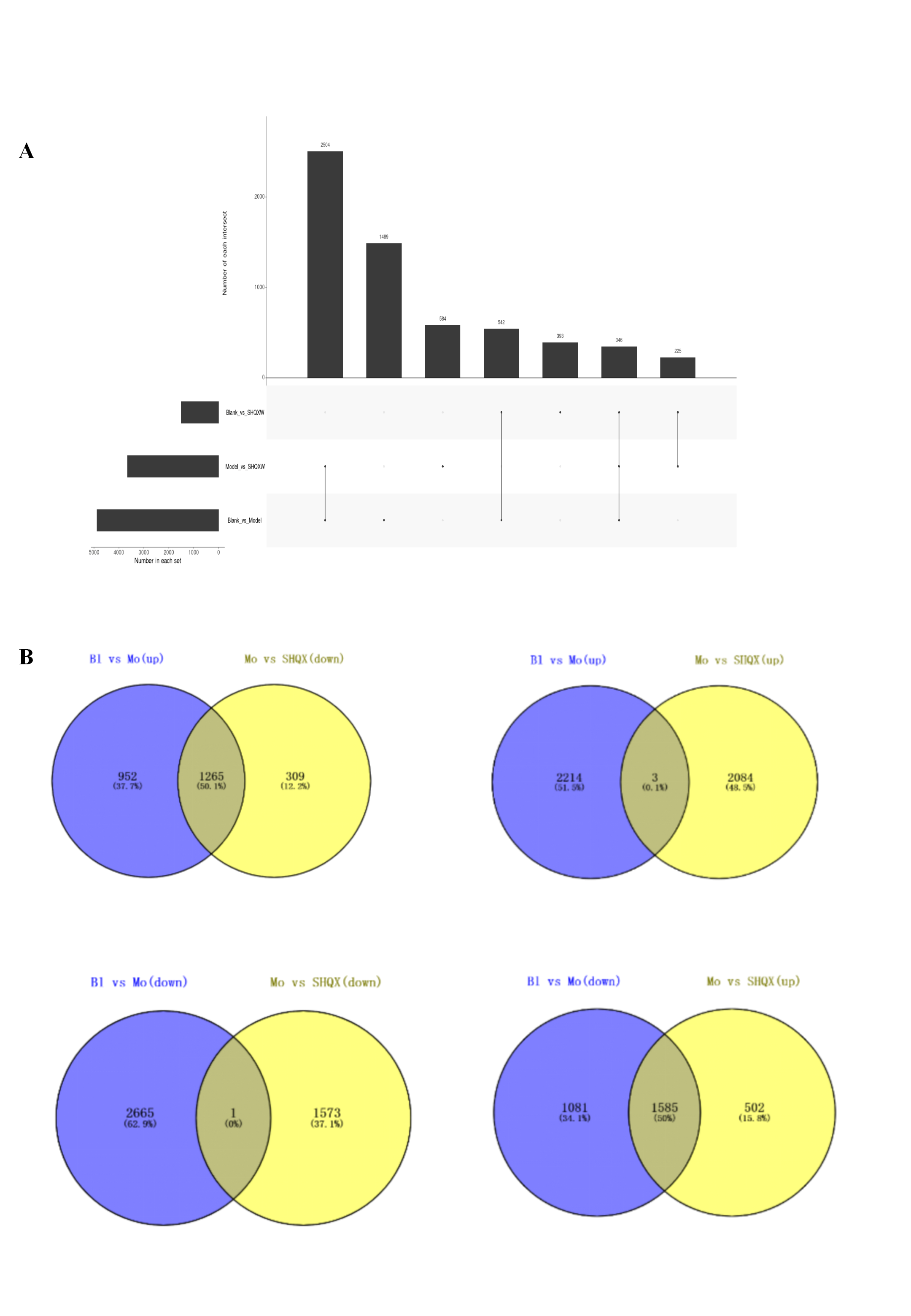
**

**Fig. S3 Differentially expressed genes (DEGs).** (A) The upset diagram of DEGs in each group. (B) The Venn diagram of DEGs in each group. Bl represents the blank group, Mo represents the model group, SHQX represents the SHQXW group.
